# FTY720 Reduces Lipid Accumulation by Upregulating ABCA1 through Liver X Receptor and Sphingosine Kinase 2 Signaling in Macrophages

**DOI:** 10.3390/ijms232314617

**Published:** 2022-11-23

**Authors:** Koki Tachibana, Kohshi Kusumoto, Mai Ogawa, Hidenori Ando, Taro Shimizu, Yu Ishima, Tatsuhiro Ishida, Keiichiro Okuhira

**Affiliations:** 1 Department of Pharmacokinetics and Biopharmaceutics, Institute of Biomedical Sciences, Tokushima University, 1-78-1, Sho-machi, Tokushima 770-8505, Japan; 2 Department of Environment and Health Sciences, Osaka Medical and Pharmaceutical University, 4-20-1, Nasahara, Takatsuki, Osaka 569-1094, Japan

**Keywords:** atherosclerosis, FTY720, ABCA1, sphingosine kinase 2, liver X receptor

## Abstract

Formation of foam cells as a result of excess lipid accumulation by macrophages is a pathological hallmark of atherosclerosis. Fingolimod (FTY720) is an immunosuppressive agent used in clinical settings for the treatment of multiple sclerosis and has been reported to inhibit atherosclerotic plaque development. However, little is known about the effect of FTY720 on lipid accumulation leading to foam cell formation. In this study, we investigated the effects of FTY720 on lipid accumulation in murine macrophages. FTY720 treatment reduced lipid droplet formation and increased the expression of ATP-binding cassette transporter A1 (ABCA1) in J774 mouse macrophages. FTY720 also enhanced the expression of liver X receptor (LXR) target genes such as FASN, APOE, and ABCG1. In addition, FTY720-induced upregulation of ABCA1 was abolished by knockdown of sphingosine kinase 2 (SphK2) expression. Furthermore, we found that FTY720 treatment induced histone H3 lysine 9 (H3K9) acetylation, which was lost in SphK2-knockdown cells. Taken together, FTY720 induces ABCA1 expression through SphK2-mediated acetylation of H3K9 and suppresses lipid accumulation in macrophages, which provides novel insights into the mechanisms of action of FTY720 on atherosclerosis.

## 1. Introduction

Atherosclerotic cardiovascular disease has been one of the leading causes of health problems in developed countries over the past decades. Macrophages, especially foam cells, play a critical role in the development and progression of atherosclerotic plaques [1]. The formation of foam cells is characterized by an imbalance of cholesterol influx and efflux and an accumulation of esterified cholesterol in cytosolic lipid droplets. Enhancing cholesterol efflux from foam cells might be an effective strategy for reducing the size of atherosclerotic plaques and thereby affording protection against atherosclerosis. It has been reported that cholesterol efflux from macrophages is mediated by several transmembrane proteins, including the ATP-binding cassette (ABC) transporters [2,3].

ATP-binding cassette transporter A1 (ABCA1) is a major regulator of cellular cholesterol and phospholipid efflux to lipid-poor apolipoprotein A-I (apoA-I), which results in the generation of high-density lipoprotein (HDL) [4,5]. The generation of HDL by ABCA1 is required to initiate the process of reverse cholesterol transport, and loss-of-function mutations in ABCA1 cause severe HDL deficiency in the plasma and a marked accumulation of lipids in peripheral tissues [6]. In human and animal models, ABCA1 mutations are positively correlated with aortic intima thickness [7] and reduce macrophage cholesterol efflux and promote atherosclerosis [8]. ABCA1 expression is regulated by the transcription factor liver X receptor (LXR), which forms a heterodimer with the retinoid X receptor (RXR) and follows the binding of specific DNA response elements within the ABCA1 promoter region to stimulate ABCA1 gene transcription [9,10,11]. This stimulation increases ABCA1 protein expression and cellular cholesterol efflux, indicating that the pharmacological upregulation of ABCA1 via LXR/RXR signaling would comprise a powerful means of stimulating cholesterol efflux in macrophages, and thus could play a significant role in preventing the development of atherosclerotic plaques.

Fingolimod (FTY720) possesses potent immunosuppressive activity and was approved by the United States Food and Drug Administration (FDA) in 2010 as the first oral immunomodulatory drug for multiple sclerosis (MS) [12]. FTY720 is mainly phosphorylated by sphingosine kinase 2 (SphK2) to generate the physiologically active form (FTY720-P), and this form acts as a high-affinity agonist of sphingosine-1-phosphate (S1P) receptors (S1PRs) [13,14]. Previous studies suggest that FTY720 has inhibitory effects on atherosclerosis, although conflicting reports exist [15,16,17,18]. The protective effects of FTY720 on atherosclerosis have been thought to be mainly due to the suppression of monocyte/macrophage emigration to atherosclerotic lesions by modulating S1P signaling [12]. On the other hand, FTY720 has been shown to increase HDL concentration in plasma, which may exert anti-atherosclerotic effects by reducing the plaque [19]. However, its effects on ABCA1 expression or lipid accumulation in macrophages have remained largely unknown. Here, we investigated the effects of FTY720 on lipid accumulation and ABCA1 expression in macrophages. We found that FTY720 decreases lipid accumulation in macrophages by increasing ABCA1 expression through the LXR and SphK2-dependent pathway, potentially in a manner independent of S1PR signaling.

## 2. Results

### 2.1. FTY720 Reduces Lipid Accumulation in J774 Mouse Macrophages

**Figure 1:**
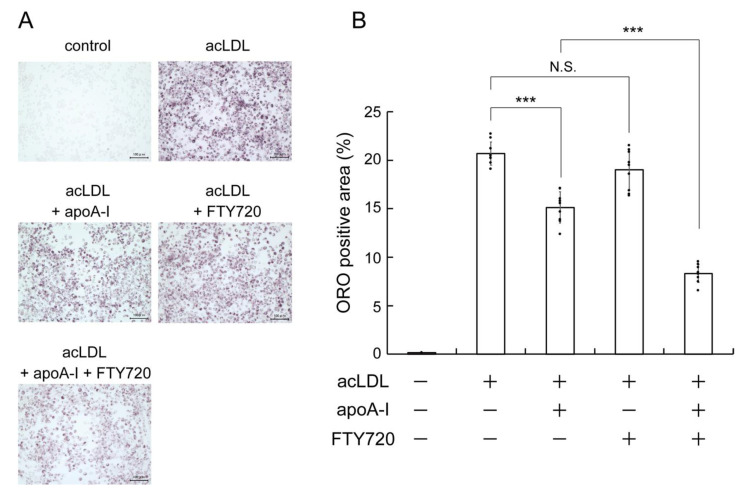
Effect of FTY720 on lipid droplet accumulation in J774 macrophages. (**A**,**B**) J774 cells were incubated with acLDL for 24 h, then treated with FTY720 (1 µg/mL = 3.25 μM) and/or apoA-I (25 µg/mL) for 24 h. Cells were fixed and stained with Oil Red O, and Oil Red O positive areas were quantified for each of the nine random images using image J. The scale bar indicates 20 μm. The magnification of each panel was ×200. Each value represents the mean ± S. D. (*n* = 9). ***, *p* < 0.001. N.S.: not significant.

We first examined whether FTY720 treatment reduces the cellular lipid accumulation in macrophage cells, which are involved in the formation of the atherosclerotic plaques. In J774 mouse macrophages, intracellular lipid droplet accumulation was observed by incubating with the acetylated low-density lipoprotein (acLDL), as determined by Oil Red O staining (Figure 1A,B, acLDL). The addition of apoA-I reduced the intracellular lipid droplets in acLDL-loaded cells (Figure 1A,B, acLDL + apoA-I). ApoA-I is known to mediate the release of intracellular lipids including cholesterol, suggesting that apoA-I actively reduces intercellular lipids by removing them out of cells [4]. FTY720 treatment had little effect on the accumulation of lipid droplets in acLDL-loaded macrophages, whereas the combination treatment of FTY720 and apoA-I significantly reduced lipid accumulation compared with apoA-I alone (Figure 1A,B, acLDL + FTY720 or acLDL + apoA-I + FTY720). These results suggest that FTY720 reduces lipid accumulation by enhancing lipid efflux from cells to apoA-I.

### 2.2. FTY720 Decreases Lipid Accumulation through Upregulating ABCA1 Expression in J774 Macrophages

**Figure 2:**
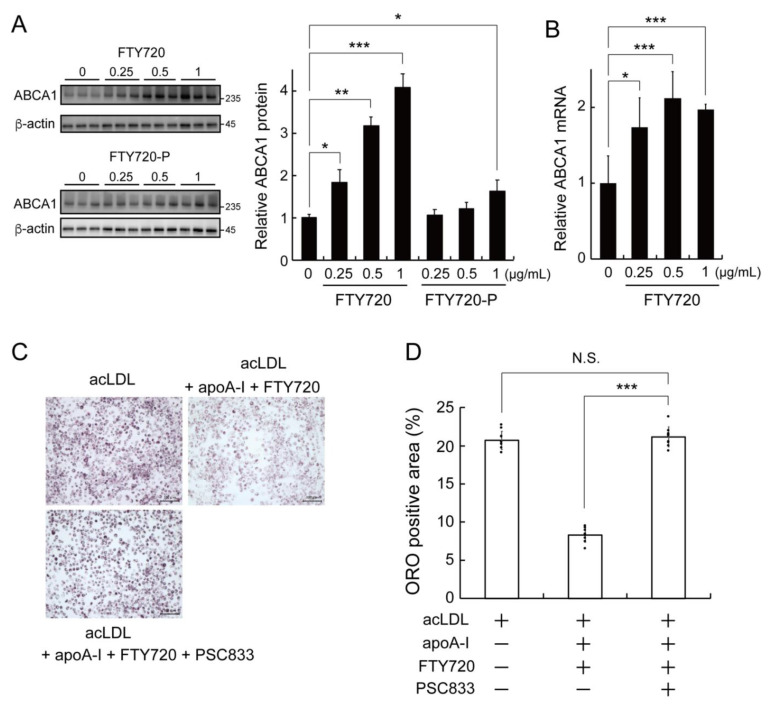
FTY720 upregulates the expression of ABCA1 and reduces lipid accumulation in J774 macrophages. (**A**) J774 cells were treated with the indicated concentrations of FTY720 (0.25, 0.5, 1.0 µg/mL = 0.81, 1.63, 3.25 μM) for 24 h. The protein expression of ABCA1 and β-actin was determined by Western blot analysis. (**B**) Cells were incubated with FTY720 for 24 h and ABCA1 mRNA levels were analyzed by RT-qPCR. The results are representative of three independent experiments. (**C**,**D**) Cells were incubated with acLDL for 24 h and then treated with FTY720 (1 µg/mL) and apoA-I (25 µg/mL) for 24 h with or without PSC833 (10 µM). Cells were fixed and stained with Oil Red O, and Oil Red O positive areas were quantified for each of the nine random images using Image J. The scale bar indicates 20 μm. The magnification of each panel was ×200. Each value represents the mean ± S. D. (*n* = 9). *, *p* < 0.05; **, *p* < 0.01; ***, *p* < 0.001. N.S.: not significant.

It is known that ABCA1 plays a major role in apoA-I-dependent lipid efflux from cells [4]. We thus examined the expression levels of ABCA1 upon FTY720 exposure in J774 macrophages. FTY720 treatment significantly upregulated ABCA1 protein level in a dose-dependent manner (0.25, 0.5, 1.0 µg/mL) (Figure 2A, FTY720). On the other hand, the phosphorylated form of FTY720, FTY720-P, increased ABCA1 protein levels only at a high concentration (1.0 µg/mL) (Figure 2A, FTY720-P), and the increasing effect of FTY720-P on ABCA1 expression was smaller than that of non-phosphorylated FTY720. RT-qPCR analysis showed that FTY720 treatment increased ABCA1 mRNA levels in J774 macrophages (Figure 2B), indicating that FTY720 treatment increased ABCA1 transcription. To investigate whether the effect of FTY720 on lipid accumulation was dependent on the expression of ABCA1, an ABCA1 inhibitor PSC833 [20] was added to FTY720-stimulated cells, and the intracellular lipid droplets were visualized with Oil Red O. As shown in Figure 2C,D, the reduction of accumulated lipid droplet by apoA-I/FTY720 treatment was significantly attenuated by the addition of PSC833. These results indicate that FTY720 reduces the intracellular lipid accumulation through increasing the expression of ABCA1.

### 2.3. LXR Activation Is Involved in the FTY720-Induced Upregulation of ABCA1 Expression in J774 Macrophages

**Figure 3:**
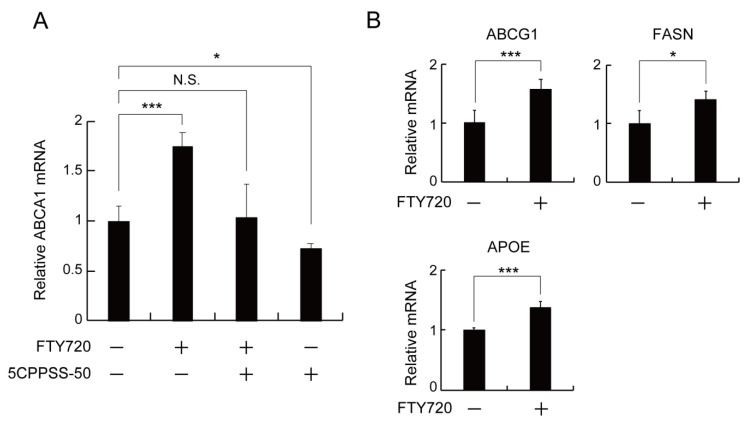
LXR is involved in the upregulation of ABCA1 expression induced by FTY720. (**A**,**B**) J774 cells were incubated with or without 1 µg/mL FTY720 (3.25 μM) and/or 0.5 µM 5CPPSS-50 (LXR antagonist) for 24 h. Relative mRNA levels were analyzed by RT-qPCR.*, *p* < 0.05; ***, *p* < 0.001. N.S., not significant. Each value represents the mean ± S.D. (*n* = 3).

A large number of studies have demonstrated that LXR activation is the key to upregulating ABCA1 expression [11]. To determine whether LXR is involved in the FTY720-mediated ABCA1 upregulation, J774 cells were treated with 5CPPSS-50, an LXR antagonist, in the presence of FTY720. As shown in Figure 3A, the treatment with 5CPPSS-50 abolished the enhancement effect of FTY720 on the ABCA1 mRNA expression. In addition, we measured mRNA levels of LXR-regulated genes, ABCG1, FASN, and APOE, in FTY720-treated J774 cells (Figure 3B). FTY720 treatment increased the mRNA expressions of ABCG1, FASN, and APOE, suggesting that FTY720 induces LXR activation and enhances target gene expressions including ABCA1.

### 2.4. SphK2 Is Involved in the FTY720-Induced Upregulation of ABCA1 Expression

FTY720 is phosphorylated in vivo to form FTY720-P, which acts as a ligand for S1PRs (mainly S1PR1) and activates diverse downstream signaling pathways [12,21]. To test whether S1PRs are involved in the regulation of ABCA1 expression, J774 macrophages were treated with S1P, a natural equivalent of FTY720-P. S1P did not increase ABCA1 expression (Supplementary Figure S1). Furthermore, a series of S1PR modulators, W146 (a selective S1PR1 antagonist), SEW2871 (a selective S1PR1 agonist), or VPC23019 (an S1PR1 and S1PR3 antagonist), also did not increase ABCA1 protein levels (Supplementary Figure S1), indicating that S1PRs and their downstream signaling may not be involved in the activation of ABCA1 by FTY720.

**Figure 4:**
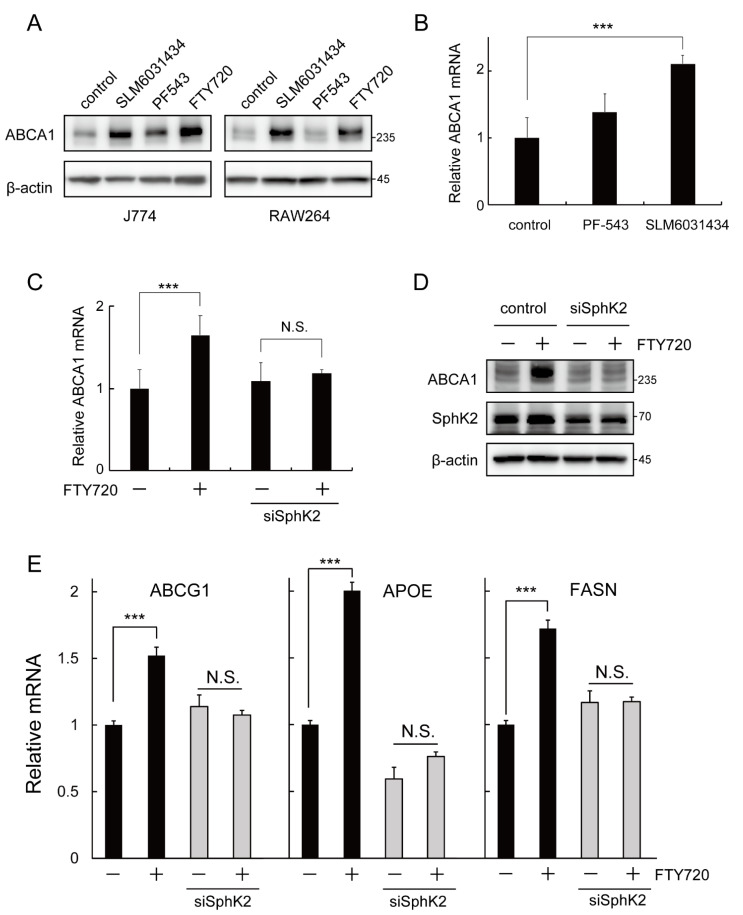
SphK2 is involved in the upregulation of the ABCA1 expression induced by FTY720. (**A**,**B**) J774 and RAW264 cells were incubated with PF-543 (40 nM) or SLM6031434 (4 µM) for 24 h. The protein expression of ABCA1 was determined by Western blot analysis. ABCA1 mRNA levels in J774 were analyzed by RT-qPCR. (**C**–**E**) RAW264 cells were transfected with negative control or SphK2 siRNA and then treated with FTY720 (1 µg/mL = 3.25 μM) for 24 h. ABCA1, ABCG1, APOE, and FASN mRNA levels were analyzed by RT-qPCR. The protein expression of ABCA1, SphK2, and β-actin was determined by Western blot analysis. Each value represents the mean ± S. D. (*n* = 3). ***, *p* < 0.001. N.S., not significant.

Previous studies reported that FTY720 is mainly phosphorylated by sphingosine kinase 2 (SphK2) and partially phosphorylated by sphingosine kinase 1 (SphK1) [13,14]. To determine whether the effect of FTY720 is associated with SphK1 or SphK2, cells were treated with either a selective SphK1 inhibitor PF-543 (Ki = 3.6 nM) [22] or a selective SphK2 inhibitor SLM6031434 (Ki = 0.4 µM) [23] and the ABCA1 protein and mRNA levels were measured. SLM6031434 significantly increased the protein expression of ABCA1 in J774 and RAW264 macrophages, while PF-543 moderately induced ABCA1 in J774 and did not increase the ABCA1 protein level in RAW264 (Figure 4A). In addition, the mRNA level of ABCA1 was significantly elevated by SLM6031434, while it was moderately induced by PF-543 in J774 cells (Figure 4B).

We then examined whether the effect of FTY720 was dependent on SphK2 expression. Surprisingly, the FTY720-induced upregulation of ABCA1 was abolished in SphK2-S-knockdown cells (Figure 4C,D). Similarly, SphK2 knockdown also eliminated the effect of FTY720 on the induction of LXR-target gene expressions (Figure 4E). These findings, along with Figure 4A,B, suggest that the interaction of these compounds with SphK2 may be involved in the increased expression of ABCA1 in mouse macrophages.

### 2.5. FTY720 Enhances ABCA1 Expression by Increasing Histone Acetylation via SphK2

**Figure 5:**
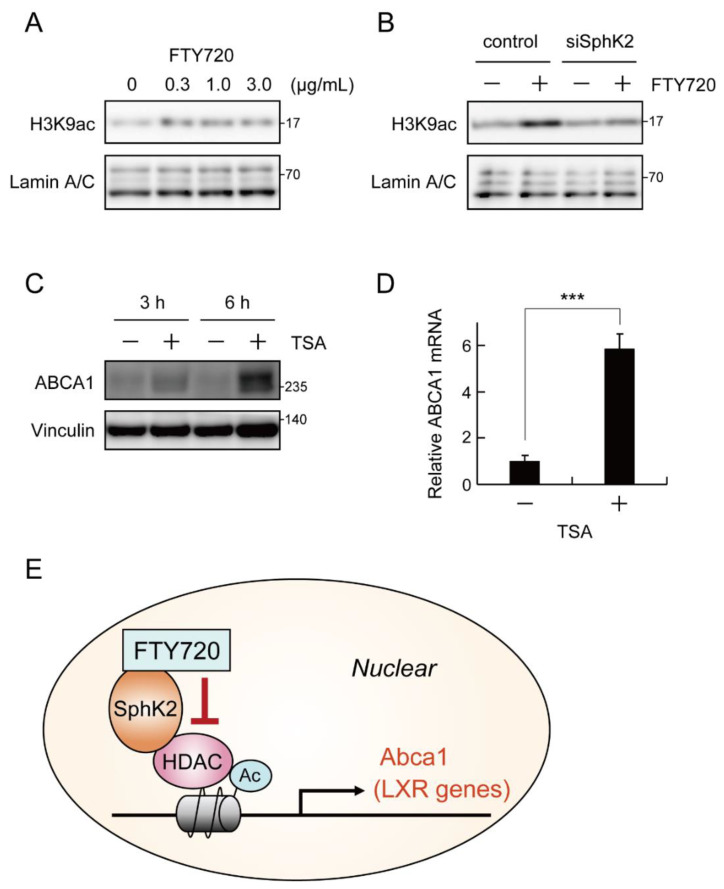
FTY720 enhances ABCA1 expression by increasing histone acetylation via SphK2. (**A**) RAW264 cells were incubated with FTY720 for 15 min at the indicated concentration. Nuclear extracts were prepared by NE-PER^TM^ Nuclear and Cytoplasmic Extraction Reagents. H3K9ac and Lamin A/C in nucleus were determined by Western blot analysis. (**B**) Cells were transfected with negative control or SphK2 siRNA for 24 h and then treated with FTY720 (1 µg/mL = 3.25 μM) for 15 min. H3K9ac and Lamin A/C in nucleus were determined by Western blot analysis. (**C**) RAW264 cells were incubated with trichostatin A (TSA; 1 μM) for the as-indicated time. The expression of H3K9ac, Lamin A/C, and ABCA1 was determined by Western blot analysis. (**D**) RAW264 cells were incubated with TSA (1 μM) for 6 h. ABCA1 mRNA levels were analyzed by RT-qPCR. Each value represents the mean ± S. D. (*n* = 3). ***, *p* < 0.001. (**E**) A model of SphK2- and HDAC-mediated promotion of ABCA1 expression by FTY720.

As shown in Figure 4, SLM6031434 and FTY720 stimulate the ABCA1 expression, and the effect of FTY720 on ABCA1 is dependent on the expression of SphK2. A study published in Science by Hait et al. showed that nuclear SphK2 binds to histone deacetylase 1 (HDAC1) and/or HDAC2 to form a co-repressor complex and epigenetically regulates transcription of target genes [24]. Therefore, we examined whether FTY720 treatment affects histone acetylation. We found that FTY720 increased acetylation of histone H3 lysine 9 (H3K9ac) (Figure 5A,B). In addition, H3K9 acetylation induced by FTY720 was attenuated in SphK2-knockdown cells (Figure 5B). Furthermore, treatment of cells with Trichostatin A (TSA), a histone deacetylase class I/II inhibitor, increased ABCA1 protein (Figure 5C) and mRNA level (Figure 5D). These findings suggest that FTY720 enhances ABCA1 expression by increasing histone acetylation via SphK2 (Figure 5E).

## 3. Discussion

FTY720 is an FDA-approved immunosuppressant as a first-in-class S1P (sphingosine-1-phosphate) receptor modulator that exerts a variety of effects, including lymphocyte depletion and anti-inflammatory responses [12,21]. It has been demonstrated that FTY720 reduces atherosclerosis when administered to LDLR^−/−^ or apoE^−/−^ mice on a cholesterol-rich Western diet [15,16]. On the other hand, little effect of FTY720 on plaque lesions has been reported in apoE^−/−^ mice on a normal laboratory diet [17] or in LDLR^−/−^ mice on a moderately hypercholesterolemic diet [18], implying that the type of diet or the degree of atherosclerosis in the animals may affect the anti-atherosclerotic action of FTY720. Thus, the effect of FTY720 on atherosclerosis in vivo has not yet been fully understood; it is generally assumed that one of the mechanisms of anti-atherosclerotic effects of FTY720 is due to the suppression of monocyte/macrophage emigration to atherosclerotic lesions by modulating S1P signaling. Meanwhile, Kan et al. recently reported that administration of FTY720 was associated with a specific increase in HDL in MS patients, suggesting that the anti-atherosclerotic effect of FTY720 may be mediated by HDL and ABCA1 [19]. However, the effect of FTY720 on lipid accumulation and ABCA1 expression in macrophages remains largely unknown. In this study, we demonstrated that FTY720 reduced lipid accumulation in macrophages (Figure 1) and that FTY720 upregulated ABCA1 expression, which may have promoted the lipid efflux from cells (Figure 2). The ABCA1-mediated lipid efflux is a process by which excess intracellular lipids are released to extracellular apoA-I to generate HDL, which is the first step in the reverse cholesterol transport [4]. Therefore, the anti-atherosclerotic effect of FTY720 may be exerted in part as a result of promoting reverse cholesterol transport through HDL.

We further showed that the upregulation of ABCA1 expression by FTY720 is mediated by LXR (Figure 3). A nuclear receptor LXR is a major regulator of several lipid-metabolism-related genes, including ABCA1 and ABCG1, and plays an important role in cholesterol homeostasis [9]. Previous studies have shown that an LXR agonist T0901317 promotes lipid efflux in macrophages [25] and inhibits the progression of atherosclerosis in mice [26]. We demonstrated that the LXR antagonist blocked the upregulation of ABCA1 induced by FTY720 (Figure 3A), suggesting that FTY720 functions as an LXR stimulator.

The unphosphorylated FTY720 more efficiently increased ABCA1 expression than the phosphorylated FTY720-P (Figure 2A). FTY720-P is characterized as a high-affinity ligand for S1PRs, especially binding to S1PR1, while FTY720 has a low affinity for S1PRs [12,21]. Furthermore, S1P as well as three other S1PR modulators (agonists or antagonists) did not affect ABCA1 expression (Supplementary Figure S1), suggesting that S1PRs and downstream signals are not involved in the mechanism of the induction of ABCA1 expression by FTY720. The key enzyme mediating FTY720 phosphorylation is SphK2 [13,14], which is known to be expressed in several organelles, including the nucleus [27]. Interestingly, nuclear SphK2 has been reported to bind to HDAC1/2 to form a co-repressor complex and regulate transcription of target genes [24,28]. For instance, FTY720 upregulated the protein expressions of Niemann–Pick type C1 and C2 in NIH3T3 fibroblasts, and treatment with siSphK2 abolished the effect of FTY720 [29]. Indeed, our data showed that the effect of FTY720 on the induction of ABCA1 expression was lost in SphK2-knockdown cells (Figure 4C,D), indicating that the FTY720-induced ABCA1 upregulation is dependent on SphK2 expression. On the other hand, SphK2 inhibitor SLM6031434 increased ABCA1 protein and mRNA expression levels, as shown in Figure 4A,B, which appears to be inconsistent with the results in Figure 4C,D. Although it has been reported that high concentrations of FTY720 (>30 µM) may inhibit SphK2 enzymatic activity [13], it is unlikely that FTY720 at the concentration used in this study (3.25 µM) would inhibit SphK2 activity. Therefore, FTY720-induced ABCA1 upregulation may not be directly related to the enzymatic inhibition of SphK2. Given that FTY720 and SLM6031434 bind to SphK2, it is possible that the binding of these compounds to SphK2 increases ABCA1 expression. Based on the above, we assume that the ABCA1-increasing effect of FTY720 may be primarily dependent on transcriptional activation by SphK2 with binding ligands, rather than on enzymatic inhibition of SphK2.

Recent studies reported that FTY720 treatment caused HDAC inhibition in MCF7 cells [30] and that administration of FTY720 increased histone acetylation in the liver of NASH model mice [31]. We showed here that FTY720 induced histone acetylation in an SphK2-expression-dependent manner (Figure 5A,B). Furthermore, treatment with HDAC1/2 inhibitor TSA markedly increased ABCA1 expression (Figure 5C,D). These results strongly suggest that FTY720 enhances ABCA1 expression by inhibiting histone deacetylation. We also found that TSA treatment enhanced mRNA expression of LXR-targets ABCG1 and APOE, but not LXRA and FASN (Supplementary Figure S2). TSA is known to inhibit a wide range of HDACs belonging to class I and II. It may suggest that the FTY720–SphK2 complex regulates LXR and ABCA1 expression using only specific HDAC species, although the detailed mechanism by which SphK2 regulates LXR activation remains to be elucidated. Genetic disruption of SphK2 in mouse models has been reported to exacerbate atherosclerosis [32], thus epigenetic regulation of ABCA1 and LXR target genes by SphK2 may be the key to understanding the anti-atherogenic mechanism of FTY720.

In conclusion, this study demonstrates that FTY720 reduces lipid accumulation through the upregulation of ABCA1 expression via LXR and SphK2 in mouse macrophages. These findings offer a new perspective on the use of FTY720 in the treatment of atherosclerosis.

## 4. Materials and Methods

### 4.1. Materials and Reagents

RPMI-1640 medium, penicillin and streptomycin solution, 4% paraformaldehyde, and 5CPPSS-50 were purchased from FUJIFILM Wako Pure Chemical Corporation (Osaka, Japan). Fetal bovine serum (FBS) was purchased from Gibco (Grand Island, NY). ISOGEN II reagent was purchased from Nippon Gene (Toyama, Japan). Lipofectamine™ RNAi Max transfection reagent was purchased from Invitrogen (Carlsbad, CA, USA). One Step TB Green Prime Script RT-PCR Kit and CellAmp™ Direct RNA Prep Kit for RT-PCR were purchased from Takara Bio (Shiga, Japan). Mouse monoclonal anti-β-actin antibody (M2), mouse monoclonal anti-vinculin antibody, protease inhibitor cocktail, and Oil Red O solution were purchased from Sigma-Aldrich, Merck KGaA (Darmstadt, Germany). Rabbit monoclonal anti-acetyl histone H3 (Lys9) antibody was purchased from Cell Signaling technology (Danvers, MA, USA). Rabbit polyclonal anti-SphK2 antibody was purchased from Proteintech (Rosemont, IL, USA). Lamin A/C antibody was purchased from Santa Cruz Biotechnology (Dallas, TX, USA).Horseradish peroxidase (HRP)-conjugated goat polyclonal anti-rat IgG antibody were purchased from Jackson ImmunoResearch Laboratory Inc. (West Grove, PA, USA). HRP-conjugated goat polyclonal anti-mouse IgG antibody were purchased from Bio-Rad Laboratories (Hercules, CA, USA). PSC833 was purchased from Novartis Pharma (Basel, Switzerland). FTY720, FTY720-P, W146, SEW2871, VPC23019, FTY720, PF-543, and SLM6031434 were purchased from Cayman Chemical (Ann Arbor, MI, USA). Rat monoclonal anti-ABCA1 antibody was kindly provided by Dr. S. Abe-Dohmae (Chubu University). Acetylated low-density lipoprotein (acLDL) was kindly provided by Dr. M. Tsujita (Nagoya City University).

### 4.2. Cell Culture and siRNA Transfection

Mouse macrophage cell line J774 and RAW264 (Japan Health Sciences Foundation) were cultured in RPMI-1640 (10% FBS, 100 µg/mL streptomycin and 100 U/mL penicillin) at 37 °C in a 5% CO_2_ humidified atmosphere. Cells were transfected using Lipofectamine ™ RNAi Max following the manufacturer’s protocol. siRNA targeting SphK2 (sense 5’-UUAUUGCAUAGACCUUUUCAA-3’ and antisense 5’-GAAAAGGUCUAUGCAAUAAAG-3’) was purchased from Sigma-Aldrich. A negative control siRNA was purchased from RNAi Inc. (Tokyo, Japan).

### 4.3. Evaluation of Lipid Accumulation by Oil Red O Staining

J774 macrophages were seeded on 12-well plates (2.5 × 10^5^ cells/well). The cells were incubated with 50 µg/mL acLDL for 48 h. Subsequently, the cells were treated for 24 h with FTY720, apoA-I, and/or PSC833, as indicated. The cells were washed with PBS and incubated with 4% paraformaldehyde for 10 min. After rinsing with 60% isopropanol (Nacalai Tesque, Kyoto, Japan), cells were incubated with Oil Red O solution for 10 min, and then washed once with 60% isopropanol. Stained cells were then observed under a fluorescence microscope (BZ-9000, Keyence, Osaka, Japan). Nine images of each treatment were used to calculate the Oil Red O positive area using Image J software.

### 4.4. Reverse Transcription-Quantitative Polymerase Chain Reaction (RT-qPCR)

Total RNA was extracted using a CellAmp™ Direct RNA Prep Kit from cells grown in 96-well plates using the manufacturer’s instruction. mRNA expression were quantified using a StepOnePlus™ Real-Time PCR System (Applied Biosystems, Foster City, CA, USA) with a One Step TB Green ^®^ PrimeScript™ RT-PCR kit (Takara Bio Inc., Osaka, Japan, RR066A) and the following gene-specific primers: GAPDH sense 5’-TGCCATCAACGA CCCCTTCA-3’ and antisense 5’-TGACCTTGCCCACAGCC TTG-3’; ABCA1 sense 5’-TCCAGGCCAGTACGGAATTC-3’ and antisense 5’-ACTTTCCTCGCCAAACCAGTAG-3’; LXRα sense 5’-TCTGCGGTGGAGCTGTGGAA-3’ and antisense 5’-TGACGCTGGGCGGAAGAAT-3’; apoE sense 5’-TCTGCGGTGGAGCTGTGGAA-3’ and antisense, 5’-TGACGCTGGGCGGAAGAAT-; and 3’ ABCG1 sense 5’-TCTGCGGTGGAGCTGTGGAA-3’ and antisense 5’-TGACGCTGGGCGGAAGAAT-3’. PCR was performed under the following conditions: denaturation at 50 °C for 2 min, followed by 38 cycles of 95 °C for 15 s and 60 °C for 1 min. Gene expression was normalized to internal controls and fold changes were calculated using relative quantification (2-ΔΔCq). The amplification specificity was checked by melting curve analysis.

### 4.5. Western Blot Analysis

Cells were lysed on ice with RIPA buffer (20 mM Tris-HCl, 1% Triton X-100, 0.1% SDS, 1% sodium deoxycholate, 150 mM NaCl) containing protease inhibitors. Nuclear extract was prepared by NE-PER^TM^ Nuclear and Cytoplasmic Extraction Reagents (Thermo Scientific, Rockford, IL, USA). Equal amounts of protein extracts were separated by SDS-PAGE and transferred to PVDF membranes. Following blocking in 4% skim milk in water at room temperature for 1 h, the membranes were incubated overnight at 4 °C with the appropriate primary antibodies (ABCA1 or β-actin). The following day, membranes were incubated with the respective secondary antibodies for 1 h at room temperature after rinsing three times with TBS-T buffer. ImageQuant LAS 4000 system (Cytiva, Tokyo, Japan) was used to capture the protein bands. The optical density of each band was quantitated using Image J software and normalized to the intensity of β-actin.

### 4.6. Statistical Analysis

The experiments were performed three or more times. The data are presented as the mean ± standard deviation (S.D.). The statistical significance of differences between groups was analyzed with Student’s *t*-test or one-way ANOVA with Tukey’s post hoc test using GraphPad Prism 5.0 software. Values of *p* < 0.05 were considered to indicate statistically significant differences.

## Data Availability

The data presented in this study are available upon request from the corresponding author.

## Supplementary Materials

The following supporting information can be downloaded at https://www.mdpi.com/article/10.3390/ijms232314617/s1.
