# Supplementary material for: FTY720 Reduces Lipid Accumulation by Upregulating ABCA1 through Liver X Receptor and Sphingosine Kinase 2 Signaling in Macrophages
Source: Int J Mol Sci. 2022 Nov 23;23(23):14617. doi: 10.3390/ijms232314617 (PMC9740778; doi:10.3390/ijms232314617)
Supplement: Supplementary file 1 [file ijms-23-14617-s001.zip › ijms-2026269-supplementary.pdf]

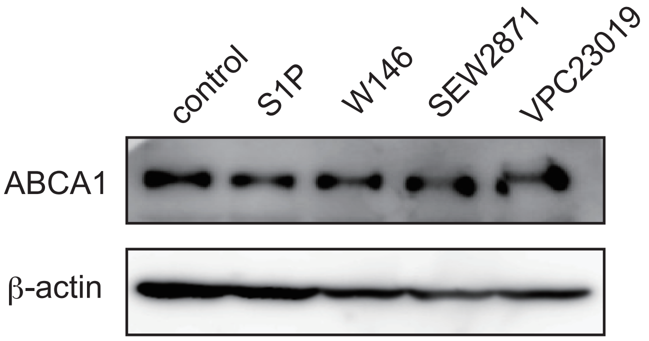

Supplemental Figure S1. SIP receptors may not involved in the FTY-induced ABCA1 upregulation.
J774 cells were incubated with S1P (3 μM), W146 (1 μM), SEW2871 (1 μM) or VPC23019 (1 μM) for 24 h. ABCA1 and β-actin were analyzed by western blotting.

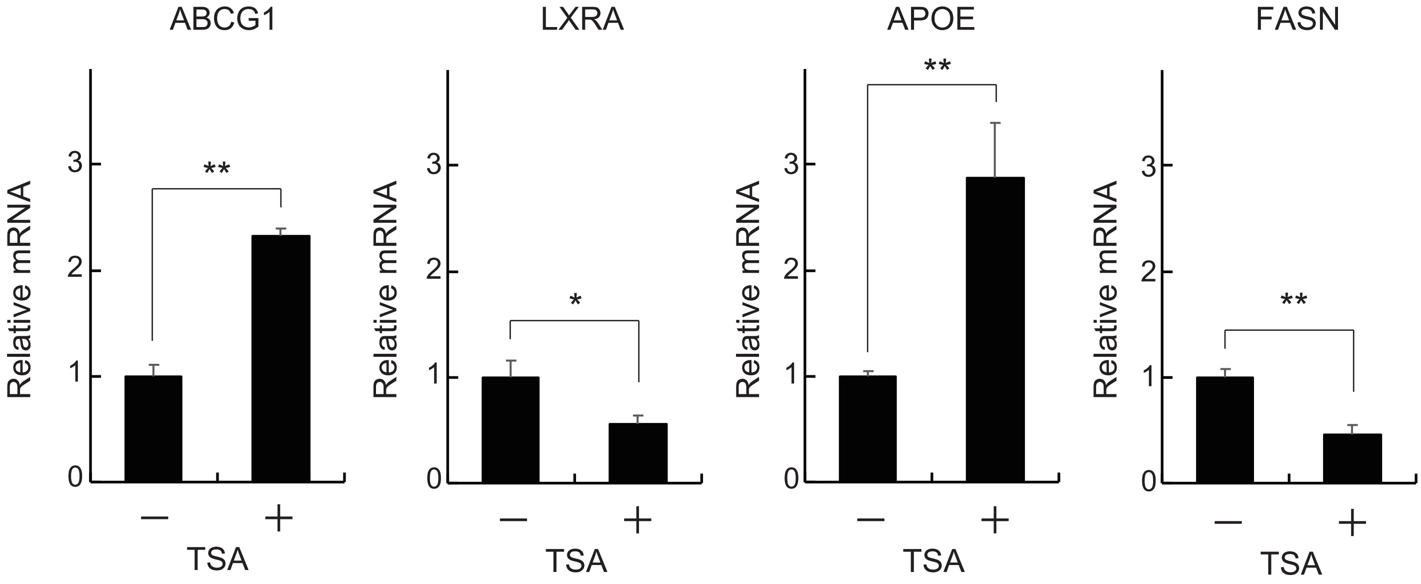

Supplemental Figure S2. Changes of LXR-tarrgeted genes by Tricostatin A (TSA) treatment.
RAW264 cells were incubated with TSA (1 μM) for 6 h. ABCG1, LXRA, APOE, FASN mRNA levels were analyzed by RT-qPCR. Each value represents the mean ± S. D. (n = 3). P < 0.001.
